# Circulating microRNA Profiles in Acute Spinal Cord Injury: Evidence for Distinct Plasma Signatures Compared with Polytrauma Patients

**DOI:** 10.3390/ijms262210954

**Published:** 2025-11-12

**Authors:** Jason-Alexander Hörauf, Miriam Saenger, Philipp Störmann, André El Saman, Ingo Marzi, Dirk Henrich, Liudmila Leppik, Cora Rebecca Schindler

**Affiliations:** Department of Trauma Surgery and Orthopedics, University Hospital, Goethe University Frankfurt, 60596 Frankfurt am Main, Germany

**Keywords:** spinal cord injury, miRNA, biomarkers, neurotrauma, polytrauma

## Abstract

Traumatic spinal cord injury (SCI) is a devastating complication of trauma, causing long-term disability and significant socioeconomic burden. Beyond the primary mechanical insult, secondary injury cascades involving apoptosis, oxidative stress, and inflammation amplify tissue damage. MicroRNAs (miRNAs) regulate these processes at the post-transcriptional level, yet data on circulating miRNAs in human SCI remain scarce. This study aimed to characterize acute plasma miRNA expression patterns in isolated traumatic SCI that may indicate SCI-specific signatures. Plasma was collected from five SCI patients at admission and 48 h post-injury and five healthy controls (HCs), and next-generation sequencing (NGS) was performed on plasma RNAs. Differentially expressed miRNAs were identified, and selected candidate miRNAs were validated by droplet digital PCR (ddPCR) in an expanded cohort of SCI patients, polytrauma patients without neurotrauma (PT), and HC (each *n* = 8). Pathway enrichment and validated target analysis were performed to assess biological relevance of candidate miRNAs. At emergency room admission, 46 miRNAs were differentially expressed in SCI plasma (18 upregulated, 28 downregulated). By 48 h, a global downregulation was observed, with 47 miRNAs significantly decreased compared with HC. ddPCR validation revealed markedly stronger suppression of miR-182-5p, miR-190a-5p, miR-144-5p, and miR-30c-5p expression levels in SCI compared with PT. Pathway analysis indicated enrichment of mitochondrial oxidative phosphorylation pathways, and target prediction suggested that the identified miRNAs may be linked to neuroprotective and regenerative functions. Our findings demonstrate early and profound alterations in circulating miRNAs after acute SCI. The downregulation of the identified miRNAs may reflect maladaptive changes that promote neuroinflammation and hinder axonal regeneration, although the exact functional consequences remain to be clarified. These data suggest that circulating miRNAs could hold promise as diagnostic and prognostic biomarkers and, potentially, as therapeutic targets to influence secondary injury processes. However, given the exploratory nature and limited sample size of this study, the findings should be validated in larger, sufficiently powered cohorts to robustly delineate differences between patient groups.

## 1. Introduction

Polytrauma (PT) remains one of the primary causes of morbidity and mortality in young adults worldwide and is associated with substantial socioeconomic consequences [1,2]. Within the spectrum of polytrauma, spinal injuries rank third in frequency after head injuries (45.9%) and chest injuries (45.7%), accounting for approximately 30% of cases [3]. Although less frequent, traumatic spinal cord injuries (SCIs) account for an estimated 250,000–500,000 new cases worldwide each year and have profound, long-term consequences for affected individuals, including chronic pain, autonomic dysfunction, and increased susceptibility to infections and metabolic complications [4,5].

The pathophysiology of SCI is complex, consisting of a two-phase injury process. The primary insult occurs at the moment of trauma, causing mechanical disruption of neural tissue and vasculature and leading to blood–spinal cord barrier breakdown [6]. This is followed by secondary injury mechanisms, driven by cascades of ischemia, demyelination, apoptosis, oxidative stress, excitotoxicity, and inflammatory responses, which exacerbate neuronal damage and impede regeneration [7].

MicroRNAs (miRNAs)—short, non-coding RNAs that regulate gene expression post-transcriptionally—could play important roles in these processes and have emerged as relevant mediators of secondary injury [8,9]. For instance, in vivo studies have shown that the antiapoptotic miR-124—which promotes functional recovery by targeting *Bax*, *caspase-9*, and *caspase-3*—was significantly downregulated 14 days post-injury [10]. Conversely, miR-711—which downregulates Ang-1/Akt signaling, a pathway crucial for reducing apoptosis, protecting the microvasculature, and improving motor function—was found to be strongly upregulated in a murine SCI model [11]. However, only limited studies have investigated miRNA alterations in human SCI [12,13].

While MRI remains the gold standard for confirming SCI, circulating miRNAs may provide complementary diagnostic and prognostic value [14]. They could enable more precise prediction of neurological recovery than standard examinations, which in the acute phase are often confounded by polytrauma, brain injury, intoxication, or sedation [15]. Current care emphasizes early surgical decompression to limit neurological deterioration, while rehabilitation is central to long-term [16,17]. Beyond their biomarker potential, circulating miRNAs may also offer insights into the molecular mechanisms driving secondary injury, thereby opening novel diagnostic and therapeutic targets.

The primary aim of this study was to identify miRNAs with altered expression in patients with isolated SCI, and, among them, to determine candidate miRNAs that may serve as SCI-specific biomarkers and offer new insights into the acute-phase response following injury.

## 2. Results

### 2.1. Patient Characteristics

Five patients with isolated SCI accompanied by spinal fractures (mean age 35.6 years, all male) and five age- and sex-matched healthy volunteers (34.4 years, all male) were included in the next-generation sequencing (NGS) analysis. Neurological impairment of these patients was classified according to the American Spinal Injury Association (ASIA) Impairment Scale as ASIA grade A (complete loss of motor and sensory function below the level of injury, *n* = 3) and grade B (preserved sensory but not motor function below the level of injury, *n* = 2). For the subsequent validation by droplet digital PCR (ddPCR), the existing cohort was expanded by three additional SCI patients (*n* = 1 ASIA grade A, *n* = 2 ASIA grade C), resulting in a total of eight SCI patients. The SCI and PT cohorts (each *n* = 8) used in validation study were comparable in age (SCI: median age 43.5 years vs. PT: 41 years), sex distribution (6 of 8 male in both groups), and injury severity (median Injury Severity Score [ISS] 25 in both groups).

Upon admission to the emergency room (ER), SCI patients showed lower inflammatory markers (interleukin-6 [IL-6]: 23 pg/mL; leukocyte count: 8.2/nL) compared with PT patients (IL-6: 60.8 pg/mL; leukocyte count: 11.3/nL), though differences did not reach statistical significance. Lactate levels at admission were also lower in the SCI group (19.5 mg/dL vs. 40.5 mg/dL, *p* = 0.078). At the scene, SCI patients had significantly lower systolic blood pressure (85 mmHg vs. 120 mmHg, *p* = 0.038) and, accordingly, more frequent vasopressor use upon ER admission (75% vs. 25%, *p* = 0.132). No significant differences were observed in hemoglobin levels at admission. No in-hospital mortality occurred in either group. Demographic and clinical characteristics of the patients are summarized in Table 1.

### 2.2. NGS-Based miRNA Expression Profiling

To explore whether circulating plasma miRNAs reflect spinal cord injury, NGS was performed to compare plasma from SCI patients collected at two time points, i.e., upon ER admission and 48 h post-injury, thereby capturing the acute phase of SCI and healthy controls (HC).

**Figure 1:**
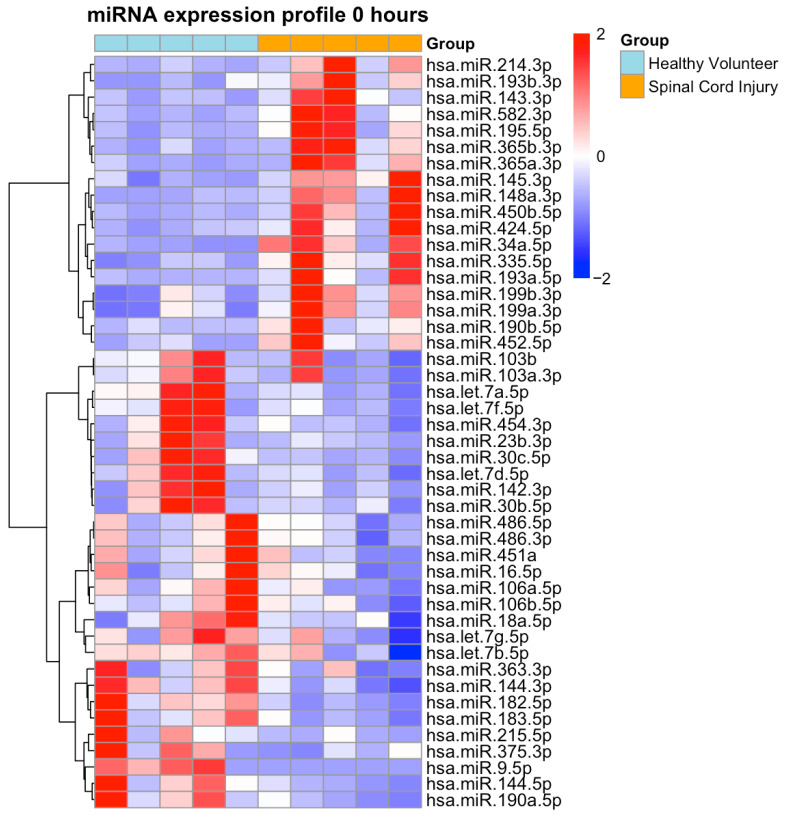
Heatmap of Differentially Expressed Plasma miRNAs at Emergency Room Admission. Heatmap illustrates expression profiles of miRNAs in patients with isolated traumatic spinal cord injury (SCI; orange) at emergency room admission (0 h) and healthy volunteers (light blue). A total of 46 miRNAs were differentially expressed (18 upregulated, 28 downregulated; DESeq2, Benjamini–Hochberg adjusted *p* < 0.05, FDR correction). Data are log_2_-transformed, centered, and scaled per miRNA. Rows and columns are hierarchically clustered (Euclidean distance). Red indicates up- and blue indicates down-regulation in SCI.

At ER admission, a total of 46 miRNAs were differentially expressed in SCI plasma samples, including 18 upregulated and 28 downregulated miRNAs (Figure 1, Tables S2 and S3).

**Figure 2:**
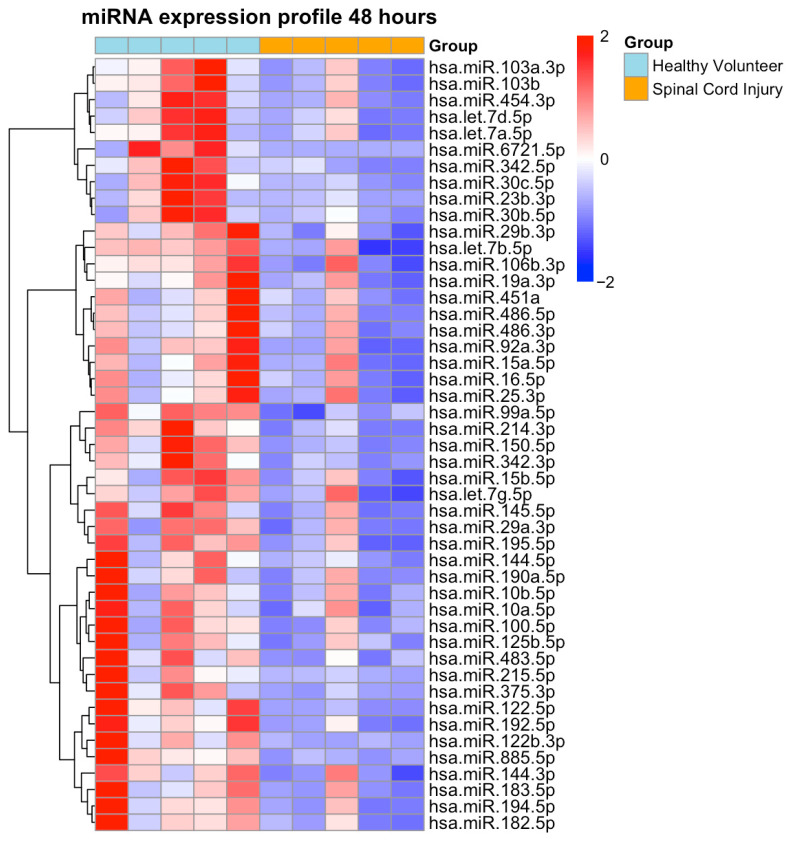
Heatmap of Differentially Expressed Plasma miRNAs at 48 h. Heatmap depicts plasma miRNA expression profiles in patients with isolated traumatic spinal cord injury (SCI; orange) at 48 h post-injury compared with healthy volunteers (light blue). A total of 47 miRNAs were significantly downregulated (DESeq2, Benjamini–Hochberg adjusted *p* < 0.05, FDR correction). Expression values were log_2_-transformed, centered, and scaled per miRNA. Rows and columns were hierarchically clustered using Euclidean distance. Red indicates up- and blue indicates down- regulation in SCI.

At 48 h post-injury, a marked global downregulation of miRNA expression was detected, with 47 differentially expressed miRNAs showing significantly reduced levels compared to healthy controls (Figure 2, Table S4). Notably, no miRNAs were found to be significantly upregulated at this time point.

Considering the within-group variability observed among SCI patients, an intraindividual analysis was performed comparing miRNA expression at 0 h (ER) and 48 h. In total, 23 miRNAs were significantly dysregulated compared to healthy controls at both time points. For these 23 overlapping miRNAs, Δ(log_2_ expression) = 48 h − 0 h was calculated. The results are presented as paired line plots (Supplementary Figure S1), illustrating the direction and magnitude of change for each miRNA per patient. The majority of miRNAs exhibited a consistent downward trend, indicating a recurring pattern of post-injury downregulation rather than random interindividual variation.

### 2.3. Over-Representation-Analysis Using Reactome

To further explore the functional relevance of the dysregulated plasma miRNAs, an over-representation analysis (ORA) was conducted using the Reactome database. This approach enables the systematic mapping of miRNAs to curated biological signaling pathways and cellular processes [18].

**Figure 3:**
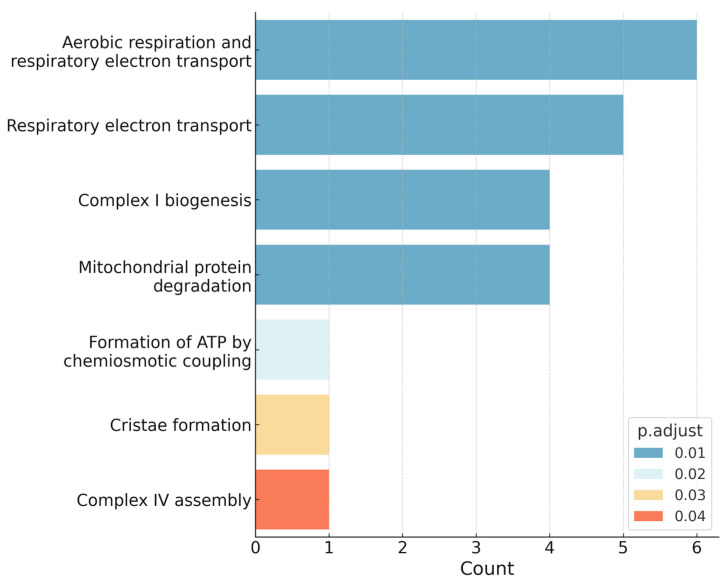
Enriched pathways in SCI identified via over-representation-analysis using Reactome. The bar plot illustrates the most enriched biological pathways associated with differentially expressed miRNAs in SCI patients compared with healthy controls. Bar length indicates the number of mapped genes, while bar color reflects the adjusted *p*-value (significance level). Mitochondrial processes, particularly oxidative phosphorylation (OXPHOS) and electron transport chain components, were among the top enriched pathways.

As compared with healthy controls, several pathways were significantly enriched in SCI patients. Among them, the most enriched pathways comprised key mitochondrial processes, including the electron transport chain within the oxidative phosphorylation (OXPHOS) system, overall oxidative phosphorylation, and the mitochondrial complex I assembly pathway of the OXPHOS system (Figure 3).

### 2.4. NGS-Based Selection of Candidate miRNAs

Next, we investigated if the most dysregulated miRNAs identified by NGS are specific to SCI. To identify miRNAs, which expression was altered the most, we applied a ranking approach accounting both the magnitude of expression change (fold change) and statistical significance (*p*-value) (Tables S2–S4). At ER admission (0 h), the five most upregulated (hsa-miR-34a-5p, hsa-miR-335-5p, hsa-miR-193a-5p, hsa-miR-450b-5p, and hsa-miR-582-3p) and the five most downregulated (hsa-miR-144-5p, hsa-miR-30c-5p, hsa-miR-182-5p, hsa-miR-215-5p, and hsa-miR-190a-5p) miRNAs were selected for further analysis. At 48 h post-injury, only the most downregulated miRNAs (hsa-miR-122-5p, hsa-miR-215-5p, hsa-miR-375-3p, hsa-miR-150-5p, and hsa-miR-885-5p) were considered, as none of miRNAs were upregulated at this time point. Overall, 14 candidate miRNAs (miR-215-5p was markedly dysregulated at both time points) were chosen for downstream analysis.

### 2.5. ddPCR Validation of Candidate miRNAs and Assessment of SCI Specificity

**Figure 4:**
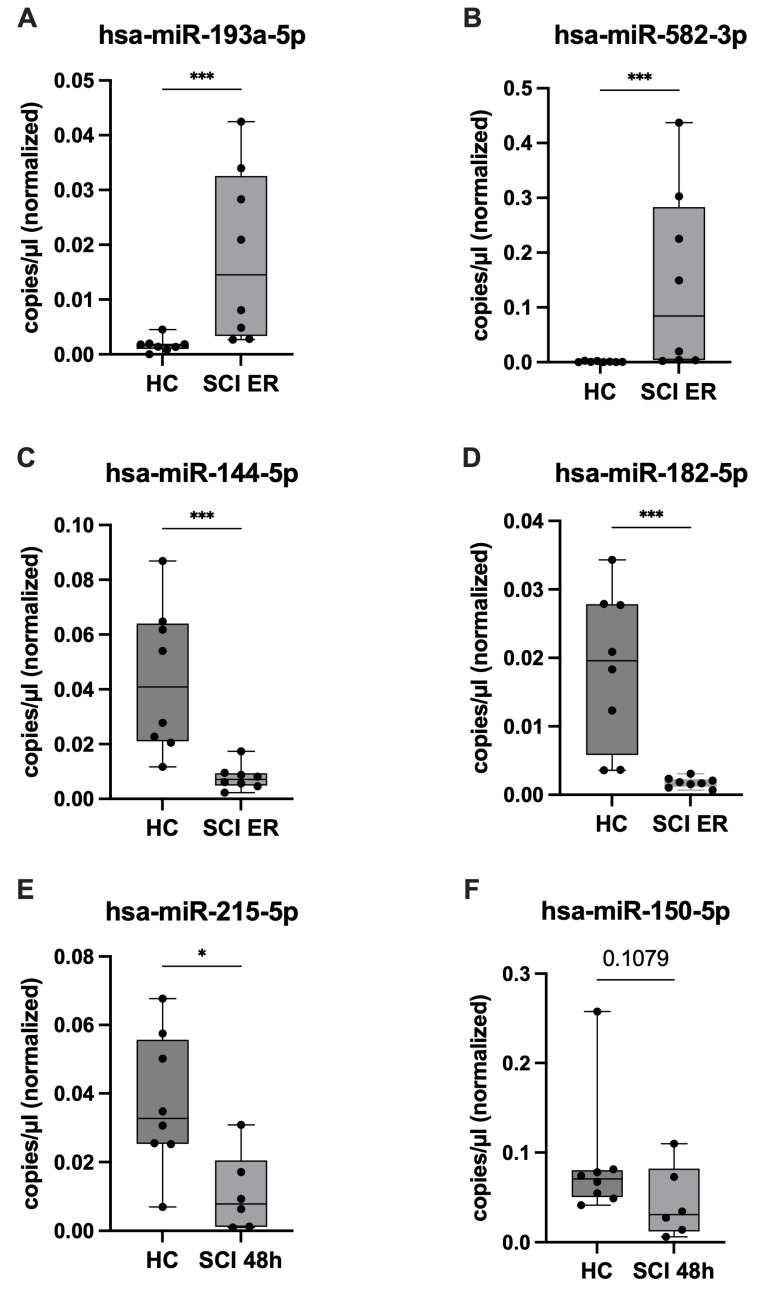
Validation of differential expression of selected plasma miRNAs by ddPCR. Differentially expressed miRNAs identified by NGS were validated using ddPCR. Shown are the two most dysregulated miRNAs per group: (**A**,**B**) expression of hsa-miR-193a-5p and hsa-miR-582-3p was significantly upregulated in SCI patients at emergency room (ER) admission compared with healthy controls (HC); (**C**,**D**) hsa-miR-144-5p and hsa-miR-182-5p were significantly downregulated in SCI patients at ER compared with HC; At 48 h post-injury, expression of hsa-miR-215-5p (**E**) was significantly decreased in SCI patients compared with HC, while hsa-miR-150-5p (**F**) showed a marked reduction. Expression differences are displayed as boxplots (median, interquartile range, whiskers), with individual values shown as dots. The *y*-axis represents concentration of miRNAs (copies/µL), normalized to the concentration of the spike-in miRNA. Group sizes were SCI (*n* = 8 at ER; *n* = 6 at 48 h) and HC (*n* = 8). Statistical significance was assessed using the Mann–Whitney U test (* *p* < 0.05, *** *p* < 0.001).

To validate the NGS results, expression levels of the candidate miRNAs were quantified by ddPCR in SCI and HC patients and compared. The results of this analysis confirmed the NGS findings for eight miRNAs (Table 2). Three additional miRNAs (hsa-miR-30c-5p, hsa-miR-375-3p, and hsa-miR-150-5p) exhibited concordant trends toward dysregulation in SCI patients, although these changes did not reach statistical significance. Therefore, these 11 candidate miRNAs were selected for further analyses to assess SCI specificity. Representative expression patterns are illustrated in Figure 4: hsa-miR-193a-5p and hsa-miR-582-3p were 10-fold and 113-fold upregulated, respectively, in SCI patients at ER compared to healthy controls (Figure 4A,B), whereas hsa-miR-144-5p and hsa-miR-182-5p were approximately 6-fold and 10-fold downregulated (Figure 4C,D). At 48 h after injury, hsa-miR-215-5p and hsa-miR-150-5p were reduced by about 4-fold and 2-fold, respectively, compared with controls (Figure 4E,F). The expression levels of the remaining validated miRNAs in the SCI and healthy control cohorts are summarized in Table 2.

**Figure 5:**
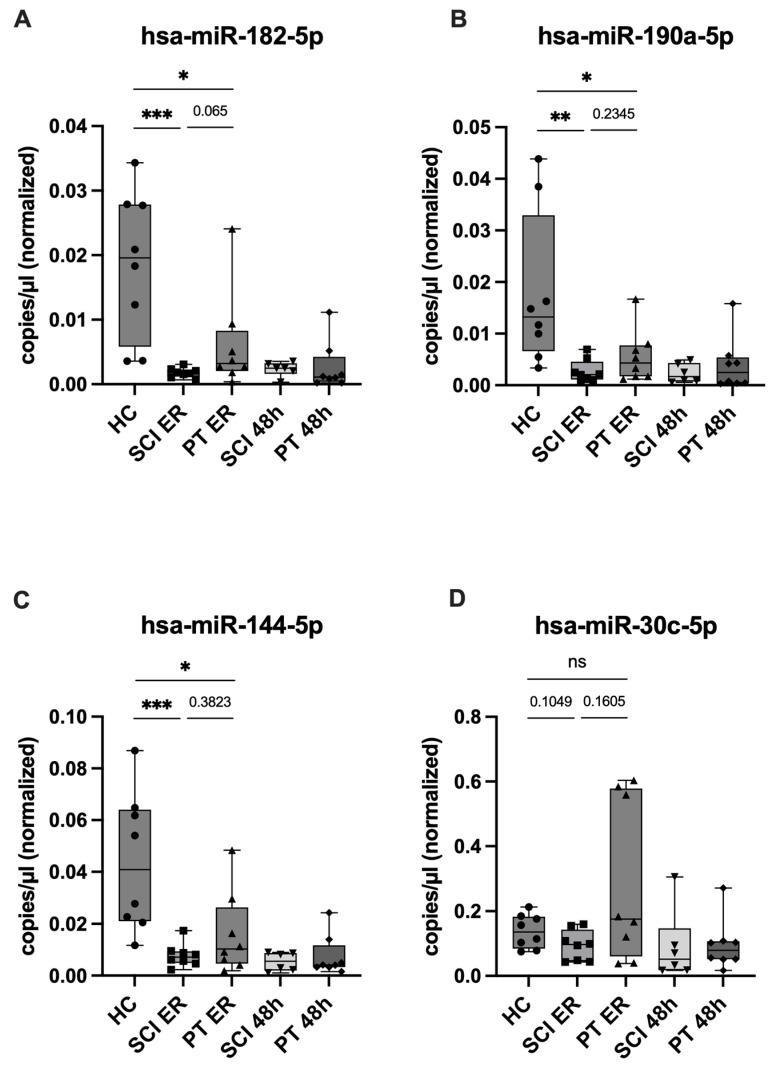
SCI-specificity of the candidate miRNAs, ddPCR expression analysis. Expression levels of candidate miRNAs measured using ddPCR at both time points in SCI and PT patients as well as in healthy controls. (**A**–**C**) Expression of hsa-miR-182-5p, hsa-miR-190a-5p, and hsa-miR-144-5p was downregulated in both trauma groups compared with HC, with a trend toward stronger suppression in SCI; and (**D**) hsa-miR-30c-5p, showed opposing regulation in SCI (downregulated) and PT (upregulated) compared with HC. Expression differences are displayed as boxplots (median, interquartile range, whiskers), with individual values shown as symbols, with different shapes representing the respective study groups. (*n* = 8). Statistical significance was assessed using the Mann–Whitney U test (* *p* < 0.05, ** *p* < 0.01, *** *p* < 0.001, ns = not significant), with *p*-values indicating group comparisons.

In the next step, to assess potential SCI-specificity, expression levels of the eleven candidate miRNAs were quantified in plasma from polytrauma patients (without neurotrauma) at ER and 48 h post-injury using ddPCR and compared with those from healthy controls and SCI patients. None of the tested candidates revealed a statistically significant SCI-specificity. However, several miRNAs showed notable patterns: hsa-miR-182-5p, hsa-miR-190a-5p, and hsa-miR-144-5p were downregulated in both PT and SCI patients as compared with HC, with a trend toward stronger suppression in SCI patients at ER, although without statistical significance (Figure 5A–C, Table S5). Additionally, in contrast to this pattern, hsa-miR-30c-5p displayed divergent regulation between the trauma groups: relative to control expression level, it was downregulated in SCI patients and upregulated in PT patients at the same time point. However, no significant difference was observed between its expression levels in the two trauma groups (*p* = 0.1605) (Figure 5D, Table S5).

### 2.6. miRNA Target Analysis Using miRTarBase

To assess the potential biological relevance of candidate SCI-associated miRNAs (hsa-miR-182-5p, hsa-miR-144-5p, hsa-miR-30c-5p, and hsa-miR-190a-5p), their experimentally validated targets were identified in miRTarBase [19]. Importantly, only inverse expression patterns were considered—i.e., cases where miRNAs were upregulated while their corresponding target genes were downregulated or vice versa—as these are most indicative of a potential regulatory interaction.

Target prediction analysis revealed several shared targets among the candidate miRNAs miR-30c-5p, miR-182-5p, and miR-144-5p. Specifically, *Cyclin D2* (*CCND2*) and *Forkhead box O3* (*FOXO3*) were both predicted targets for miR-30c-5p, miR-182-5p, while *Mothers against decapentaplegic homolog 4* (*SMAD4*) was a common target of miR-182-5p and miR-144-5p. Beyond these direct target matches, additional commonality was observed at the gene family level. This included members of the *B-cell-lymphoma* (*BCL*) family (*BCL9*, *Myeloid cell leukemia-1* (*MCL1*) for miR-30c-5p; *BCL2* for miR-182-5p), the *cyclin* (*CCN*) family (*CCND2* for miR-30c-5p and miR-182-5p; *CCNE1/2* for miR-144-5p), and the *SMAD* family (*SMAD4* for miR-144-5p and miR-182-5p; *SMAD1* for miR-30c-5p) (Table S6). These gene families play key roles in regulating apoptosis, cell cycle progression, and axonal growth and regeneration, suggesting that the three miRNAs may converge on shared biological pathways relevant to secondary injury following spinal cord injury. Under the applied analysis conditions, miR-190a-5p did not yield any shared or high-confidence predicted target genes.

### 2.7. Correlation of miRNA Expression with Neurological Status and Systemic Inflammation

To explore the potential diagnostic and prognostic relevance of candidate SCI-associated miRNAs, we analyzed correlations between their plasma levels at ER admission and the degree of neurological impairment assessed by ASIA grade. Among the investigated candidates, hsa-miR-182-5p showed the most pronounced negative correlation (rho = −0.52, *p* = 0.041), with lower levels observed in patients with severe impairment (ASIA A/B) and relatively higher levels in patients with milder deficits (ASIA C). Similarly, hsa-miR-30c-5p (rho = −0.48, *p* = 0.064) and hsa-miR-190a-5p (rho = −0.48, *p* = 0.062) showed moderate negative trends with ASIA grade. No relevant correlation was observed for hsa-miR-144-5p (rho = −0.32, *p* = 0.221).

To assess whether the observed miRNA alterations were associated with neuronal damage rather than inflammation, we examined correlations between candidate miRNA levels and systemic IL-6 concentrations as well as leukocyte counts. In the SCI group, no significant associations were detected between IL-6 and hsa-miR-182-5p (rho = −0.11, *p* = 0.82), hsa-miR-144-5p (rho = 0.57, *p* = 0.18), hsa-miR-30c-5p (rho = 0.64, *p* = 0.12), or hsa-miR-190a-5p (rho = 0.04, *p* = 0.94). By contrast, in the PT group, hsa-miR-190a-5p showed a positive correlation with IL-6 (rho = 0.86, *p* = 0.014). Similarly, no correlation was observed between leukocyte counts and candidate miRNAs levels in the SCI group, while a tendency toward moderate positive associations was noted for hsa-miR-182-5p (rho = 0.55, *p* = 0.16), hsa-miR-144-5p (rho = 0.55, *p* = 0.16), and hsa-miR-190a-5p (rho = 0.52, *p* = 0.18) in the PT group.

## 3. Discussion

After traumatic SCI, secondary injury mechanisms—including neuroinflammation, oxidative stress, neuronal apoptosis, glial reactivity, and axonal degeneration—drive disease progression and restrict regeneration; these pathways are orchestrated by injury-induced changes in gene expression, with miRNAs functioning as central post-transcriptional regulators [20]. In this context, the study aimed to characterize miRNA expression changes in patients with isolated SCI and to identify candidates that may serve as potential SCI-specific biomarkers, thereby advancing our understanding of early secondary injury mechanisms. We demonstrated that expression of numerous plasma miRNAs is altered during the acute phase after SCI. Moreover, several miRNAs displayed distinct expression differences compared with plasma from PT patients, suggesting a potential role associated with SCI. Most notably, hsa-miR-182-5p and hsa-miR-144-5p were markedly more suppressed in SCI patients at the time of ER admission relative to PT patients. In contrast, hsa-miR-30c-5p demonstrated an inverse expression pattern between the two trauma groups.

### 3.1. Circulating miRNAs as Potential Blood-Based Biomarkers in Acute SCI

In our study, we observed a broad deregulation of plasma miRNAs at admission (46 differentially expressed; 18 up, 28 down), followed by a global downregulation at 48 h (47 differentially expressed, all decreased). In contrast, Tigchelaar et al. reported nearly 200 differentially expressed miRNAs in cerebrospinal fluid (CSF), compared with only 19 detected in serum across days 1–5 post-injury [21]. Our findings reveal a greater degree of deregulation compared to this study, which could partly reflect inherent differences between the analyzed biofluids. Plasma is generally considered more sensitive than serum for detecting cell-free and extracellular vesicle–associated miRNAs, since coagulation during serum preparation can alter miRNA profiles through platelet activation and release [22,23]. In addition, whereas Tigchelaar et al. reported predominantly upregulated miRNAs in CSF, we observed mostly downregulation, indicating possible compartment-specific regulation. Similar suppression has been reported in animal studies and was linked to secondary degeneration and transcriptional reprogramming after SCI [20]. However, the general downregulation of plasma miRNAs may result from increased cellular uptake or accelerated hepatic and renal clearance, thereby reducing their detectable levels in circulation. This may explain the observed disappearance of early signals.

Despite these limitations, blood-based miRNAs represent highly attractive biomarker candidates. Unlike CSF—which, although closer to the site of injury, requires invasive sampling and is often impractical in unstable trauma patients—plasma offers a minimally invasive alternative that can be collected rapidly, repeatedly, and safely. This makes it particularly valuable in the emergency setting, where timely molecular insights can complement clinical evaluation and imaging-based diagnostics.

### 3.2. Distinct Regulatory Patterns of Candidate miRNAs in SCI

Among the differentially expressed candidate miRNAs, hsa-miR-182-5p, hsa-miR-144-5p, and hsa-miR-190a-5p were markedly more suppressed in SCI patients than in PT patients already at ER admission, while hsa-miR-30c-5p displayed an inverse pattern, being downregulated in SCI but upregulated in PT patients. These expression profiles suggest regulation that is more specific to neurogenic mechanisms than to systemic trauma responses, a notion supported by findings in the literature. For example, miR-182-5p was shown to decline rapidly after SCI in animal models, and its restoration improved functional recovery and reduced inflammation [24]. Similarly, miR-144-5p has been reported to be decreased after spinal cord ischemia or nerve injury, while its restoration attenuated neuroinflammation and neuropathic changes [25,26]. Knockdown of the miR-144/451 cluster led to increased cytokine release, oxidative stress, cerebral edema, and aggravated neurological deficits, thereby supporting its neuroprotective role [27]. The functional impact of miR-30c-5p appears contradictory, suggesting that its role varies depending on injury type and target pathways. In myocardial ischemia/reperfusion (I/R) models, increased miR-30c-5p aggravated tissue injury, thereby enhancing myocardial inflammation [28]. Conversely, other cardiac I/R studies demonstrated that miR-30c-5p overexpression reduced infarct size, apoptosis, and oxidative stress [29]. In spinal cord I/R injury, miR-30c-5p was found locally upregulated, driving IL-6/TNF-α release and neuronal apoptosis, whereas its knockdown exerted protective effects [30]. Similarly, after sciatic nerve injury, miR-30c-5p was elevated in nociceptive tissues and fluids, correlating with pain severity, and its inhibition delayed neuropathic pain development [31].

The early suppression of hsa-miR-182-5p and hsa-miR-144-5p after SCI in humans may indicate an acute neurogenic stress and inflammatory response. Downregulation of these miRNAs may diminish anti-inflammatory and neuroprotective signaling, thereby permitting enhanced neuroinflammation, scar formation, and impaired axonal regeneration. Such maladaptive changes emphasize the acute phase as a potential therapeutic window. In contrast, the regulation of hsa-miR-30c-5p appears highly context-dependent: while excessive expression has been linked to aggravated inflammation and tissue injury, its reduction in SCI may represent a compensatory mechanism to mitigate secondary damage.

### 3.3. Neurotrauma-Specific Regulation Beyond Systemic Trauma Responses

The distinct miRNA expression patterns indicate differences not attributable to trauma load alone, as overall injury severity was comparable between SCI and PT patients (median ISS ≈ 25). While PT patients exhibited a stronger systemic inflammatory reaction, reflected by higher IL-6 levels, leukocyte counts, and lactate levels, the downregulation of these miRNAs was more pronounced in the SCI cohort. Notably, hsa-miR-30c-5p was downregulated in SCI but upregulated in PT patients, further suggesting that its regulation is not linked to a general systemic inflammatory response. This was supported by the results of correlation analyses, which revealed no significant association between the candidate miRNA levels and systemic inflammatory markers (IL-6 and leukocyte counts) within the SCI group, but a moderate positive correlation trend in the PT group. Moreover, hsa-miR-182-5p, hsa-miR-30c-5p, and hsa-miR-190a-5p showed negative correlations with neurological impairment ASIA grade, with the lowest levels found in patients with the most severe neurological deficits. Given the small sample size, these correlations should be interpreted with caution and regarded as exploratory rather than conclusive. Nevertheless, the consistency of the observed trends across multiple miRNAs supports the hypothesis that SCI triggers distinct regulatory mechanisms not primarily driven by systemic inflammation. Specific factors inherent to SCI may contribute to this distinct regulatory pattern. The rapid onset of neurogenic shock, a critical condition resulting from autonomic dysregulation after SCI—particularly at cervical and upper thoracic levels above T6—can lead to profound hemodynamic instability and impaired perfusion, potentially shaping unique molecular responses not observed in PT patients [32]. Moreover, disruption of the blood–spinal cord barrier after SCI facilitates selective release or sequestration of miRNAs from injured neural tissue, thereby altering circulating profiles in ways that fundamentally differ from systemic trauma [6]. Overall, our findings suggest that the regulation of these miRNAs is unlikely to be driven solely by systemic trauma-induced inflammation, but instead may reflect neurogenic mechanisms and be linked to central nervous system (CNS)-specific processes triggered by SCI.

### 3.4. Oxidative Stress as a Potential Driver of Early Secondary Injury

Pathway enrichment analysis highlighted mitochondrial bioenergetic processes, including oxidative phosphorylation and electron transport chain function, as the most significantly enriched pathways in SCI plasma at admission. Oxidative stress is widely recognized as a major driver of secondary injury after SCI, with excessive ROS production triggering lipid peroxidation, Ca^2+^ imbalance, leukocyte activation, and neuronal apoptosis [33]. Mitochondria play a central role in this cascade: excitotoxic Ca^2+^ influx stimulates complex I activity, increases ATP and ROS generation, and impairs the OXPHOS system, thereby amplifying neuronal loss [34,35,36]. Consistent with this, ultrastructural studies have likewise demonstrated early (within 4–8 h post SCI) mitochondrial injury, including swelling, disrupted cristae, and eventual membrane rupture [37]. Importantly, miR-144 was shown to directly inhibit Nuclear factor erythroid 2–related factor 2 (NFE2L2/Nrf2) signaling [38], which has been associated with elevated ROS levels and aggravated neuronal injury [39]. Conversely, inhibition of miR-144 restores Nrf2 activity, reduces oxidative stress, and confers neuroprotection [40,41]. Experimental SCI models demonstrated that Nrf2 activation preserves mitochondrial potential, sustains ATP production, and reduces neuronal apoptosis [42,43].

Taken together, our findings from pathway analysis underscore oxidative stress and mitochondrial dysfunction as potential mediators of early secondary damage after SCI. The regulatory role of miR-144-5p in modulating Nrf2 signaling positions it at the intersection of oxidative stress and mitochondrial integrity, highlighting its potential pathophysiological relevance in acute SCI.

### 3.5. Candidate miRNAs Target Genes Linked to Cell Cycle Control, Apoptosis, and Regeneration

Target prediction analysis revealed that the candidate miRNAs hsa-miR-182-5p, hsa-miR-144-5p, and hsa-miR-30c-5p converge on several shared target genes with important biological functions. *FOXO3* was a common target of miR-182-5p and miR-30c-5p, while *SMAD4* and *SMAD1* were implicated in the networks of miR-182-5p, miR-144-5p, and miR-30c-5p. Beyond direct gene overlaps, convergence was evident at the gene family level, particularly among members of the *BCL* family (e.g., *BCL9*, *BCL2*) and the *cyclin*/*CCN* family (e.g., *CCND2*, *CCNE1/2*). Experimental data indicate that these miRNAs act as central nodes in pathways regulating apoptosis, inflammation, and axonal regeneration after nerve injury. Thus, miR-182-5p was found to promote axonal outgrowth and neuronal survival via suppression of *FOXO3* and activation of PI3K–AKT signaling [44]. Both miR-182 and miR-144 modulate the TGF-β/SMAD signaling pathway, which plays a critical role in fibrotic scar formation and thereby impedes axonal regeneration after SCI [45,46,47]. Notably, miR-182-5p is reduced in hypertrophic scars, and its overexpression inhibits fibroblast proliferation and collagen synthesis by targeting *SMAD4* [48]. Conversely, activation of *SMAD1* via the BMP pathway supports axonal growth and regeneration, promoting functional recovery after injury [49,50,51].

Several of our candidate miRNAs were found to target *BCL2* family members, which act as gatekeepers of mitochondrial apoptosis. Preclinical SCI models demonstrated that reduced expression of anti-apoptotic proteins such as Bcl-2 or Bcl-xL facilitates neuronal death, whereas their overexpression confers neuroprotection and functional recovery [52,53,54]. Similarly, *cyclin* family genes (e.g., *CCND*, *CCNE*) were among predicted targets, consistent with experimental findings that aberrant cell cycle activation after SCI contributes to neuronal apoptosis, glial scar formation, and microglial proliferation [55,56]. Pharmacological inhibition of cyclin-dependent kinases reduced glial reactivity, tissue loss, and improved neurological outcomes in rodent SCI models, underscoring the pathogenic role of cell cycle dysregulation [57].

This suggests that early downregulation of miR-182-5p, miR-144-5p, and miR-30c-5p may reflect a post-traumatic shift toward enhanced neuroinflammation and reduced repair capacity. These findings highlight the potential of these miRNAs not only as circulating biomarkers but also as active regulators of secondary injury mechanisms after SCI.

### 3.6. Study Limitations

The primary limitation of this study is the small sample size, which reflects the low incidence of acute spinal cord injury and the general difficulty of assembling large patient cohorts. Consequently, data on circulating miRNAs in this population remain scarce; even the largest study to date, by Tigchelaar et al., included only 29 patients [21]. In our cohort, NGS was conducted in only five patients, with ddPCR validation performed in eight, reflecting this constraint. To reduce potential confounders such as major concomitant injuries or chronic medication use, we intentionally selected a homogeneous cohort, which further narrowed the pool of eligible participants. As a result, potential sex- or age-related differences in miRNA expression could not be systematically assessed. Additionally, not all miRNA changes identified by NGS were confirmed by ddPCR. This discrepancy may be explained by the higher sensitivity of NGS compared with targeted PCR-based methods [58], but it may also reflect the limited statistical power associated with small validation cohorts.

Despite these limitations, we believe our study provides valuable new insights, particularly given the current paucity of data on circulating miRNAs in the context of acute SCI. Given its exploratory nature and limited sample size, this study should be regarded as hypothesis-generating. The observed findings warrant validation in larger, statistically powered, multicenter cohorts with extended follow-up and multiple time points to better define prognostic and mechanistic miRNA patterns and their association with long-term neurological outcomes. While CSF may offer proximity to the injury site, blood remains a practical and clinically relevant source for biomarker discovery, and future studies should also explore other small RNA species and extracellular vesicles as potential diagnostic and therapeutic targets.

## 4. Materials and Methods

### 4.1. Study Design and Patient Selection

Eight isolated spinal cord injury (SCI) patients with accompanying vertebral fractures and eight polytrauma patients (PT) without concomitant neurotrauma (no traumatic brain injury or SCI, ISS ≥ 16) admitted to the Level 1 Trauma Center at Frankfurt University Hospital (Frankfurt am Main, Germany) between 1 January 2018, and 31 December 2022 were enrolled in the study. Ethical approval was obtained from the Local Ethics Committee of the University of Frankfurt (protocol No. 89/19, approved 3 April 2019; and protocol No. 375/14, approved 18 December 2014), and all procedures were conducted in accordance with the Declaration of Helsinki and STROBE guidelines [59]. Written informed consent was obtained from all enrolled patients or their legal representatives. Only patients with a prehospital interval of less than two hours between trauma occurrence and hospital admission were included to ensure that blood sampling accurately reflected the acute phase of injury. Exclusion criteria included chronic systemic inflammatory or metabolic disease, polyneuropathy, critical illness syndrome, neurodegenerative disorders (e.g., dementia, Parkinson’s disease), chronic alcohol abuse, organic brain syndromes (e.g., epilepsy, schizophrenia), stroke, post-traumatic resuscitation, age < 18 years, and sepsis. Neurological impairment was assessed following surgical treatment and clinical stabilization in the ICU by a board-certified neurologist according to the ASIA Impairment Scale [60]. Clinical data were retrieved from electronic medical records (ORBIS, Dedalus Healthcare Group) and is shown in Table 1.

### 4.2. Blood Sample Collection

Blood samples from patients with SCI and polytrauma without neurotrauma were obtained immediately upon admission to the ER and 48 h post-injury. Samples were drawn into 7.5 mL/2.7 mL tubes (S-Monovette©, Sarstedt Inc., Nümbrecht, Germany) containing 1.6 mg EDTA K and immediately placed on ice. Plasma was separated by centrifugation at 3500× *g* for 15 min at 4 °C, aliquoted, and stored at −80 °C until further analysis. Plasma samples from healthy volunteers serving as controls (healthy controls, HC) were processed identically.

### 4.3. miRNA Next Generation Sequencing

To assess differential miRNA expression, RNA was isolated from plasma samples collected at ER and 48 h of SCI patients and HC (each *n* = 5) by mean of miRNeasy Serum/Plasma Advanced Kit (Qiagen, Hilden, Germany) following manufacturer’ protocol. Next-generation sequencing (NGS) of isolated RNAs was performed by GenXPro GmbH (Frankfurt, Germany) using the company established protocol. In brief, the library preparation involved ligation of small RNAs to adapters containing unique molecular identifiers (UMIs), followed by reverse transcription and PCR amplification. Sequencing was conducted on an Illumina NextSeq500 platform (Illumina Inc., San Diego, CA, USA) using single-end 75 bp reads. Raw sequencing data were processed by adapter and low-quality base trimming with Cutadapt (v4.6), followed by UMI-based deduplication using custom in-house scripts [61]. Quality assessment was performed with FastQC, and reads were aligned to the reference genome using Bowtie2 (v2.4.4) [62]. Transcript quantification was carried out with HTSeq (v2.0.2) [63], and differential expression analysis (DEA) was performed using DESeq2 (v1.38) [64] with Benjamini–Hochberg false discovery rate (FDR) correction, and miRNAs with adjusted *p* < 0.05 (FDR) were considered statistically significant. Comprehensive summaries of sequencing quality and processing metrics were generated with MultiQC.

### 4.4. Analysis of miRNA Expression Using Droplet Digital PCR in SCI and PT Patients

The miRNAs with the most pronounced differential expression between SCI patients and healthy controls, as identified by NGS (Table 2), were selected for further analysis based on a composite score accounted for both the magnitude of expression change (fold change) and statistical significance (*p*-value). To perform the scoring, the −log_10_ (*p*-value) was multiplied with the corresponding log_2_ (fold change) for each miRNA. miRNAs with the highest scores (each *n* = 5 up- and down- regulated miRNAs) were chosen for each time point for farther validation analysis.

For droplet digital PCR (ddPCR) validation, the initial NGS cohort of five SCI patients (3 ASIA A, 2 ASIA B) was expanded by three additional SCI patients (1 ASIA A, 2 ASIA C), resulting in a total of eight SCI patients included in the validation study. Plasma samples obtained at ER admission and 48 h post-injury from SCI and PT patients as well as from healthy controls (each *n* = 8) were analyzed using ddPCR. At 48 h post-injury, ddPCR measurements could be performed in six SCI patients due to limited sample availability.

Briefly, RNA was isolated from 200 µL plasma samples using the miRNeasy Serum/Plasma Advanced Kit (Qiagen, Hilden, Germany) according to the manufacturer’s instructions. Complementary DNA (cDNA) was generated from 6.5 µL of RNA using the miRCURY LNA RT Kit (Qiagen) following the manufacturer’s protocol. To assess reverse transcription efficiency, 0.5 µL of spike-in controls (UniSp6 and cel-miR-39-3p) were added to each reaction.

The ddPCR assays were performed on cDNA samples diluted 1:50 with RNase-free water. Each 20 μL reaction contained 9 μL of diluted cDNA, 10 μL of 2× QX200 ddPCR EvaGreen Supermix (Bio-Rad Laboratories, Hercules, CA, USA), and 1 μL of the specific miRCURY LNA miRNA PCR Assay (Qiagen N.V., Venlo, The Netherlands). Primer sequences are listed in Supplementary Table S1. According to the manufacturer’s instructions, 70 μL of droplet generation oil for EvaGreen were added, and droplets were generated using the QX200 Droplet Generator (Bio-Rad). PCR amplification was carried out in a PTC Tempo Deep Well Thermal Cycler (Bio-Rad) with the following program: 95 °C for 5 min; 40 cycles of 95 °C for 30 s (ramp 2 °C/s) and 51 °C for 1 min (ramp 2 °C/s); followed by 5 min at 4 °C and 5 min at 90 °C. Droplets were read on the QX200 Droplet Reader, and data were analyzed with QuantaSoft Analysis Pro software version 2.1 (Bio-Rad). Results are expressed as copies/μL, and values for each miRNA were normalized to the values of spike-in cel-miR-39.

### 4.5. miRNA Target Prediction and Pathway Analysis

Putative target genes of the selected differentially expressed miRNAs were predicted using the miRDB database (version 6.0; https://mirdb.org). For target validation, only inverse expression patterns were considered, i.e., upregulated miRNAs with downregulated target genes or downregulated miRNAs with upregulated target genes, indicating a potential regulatory interaction.

In a separate analysis, pathway enrichment analysis was performed using Reactome Pathway database (version 91; https://reactome.org/) to identify biologically relevant signaling cascades associated with the observed miRNA alterations.

### 4.6. Statistical Analysis

Clinical characteristics of patients values are presented as median and interquartile range (IQR) or percentage, as appropriate. *p*-values were calculated using the Mann–Whitney U test for continuous variables and Fisher’s exact test for categorical variables.

Heat maps of NGS data were generated in R (version 4.4.0; R Foundation for Statistical Computing, Vienna, Austria) using the ggplot2 (version 3.4.4) and pheatmap (version 1.0.12) packages. Data were log_2_-transformed and z-score normalized across samples prior to visualization. Hierarchical clustering was applied using Euclidean distance and Ward’s D2 linkage method. Results of miRNA expression analysis are presented as box plots of the median in diagrams. For comparisons between two groups, the Mann–Whitney U test was used. For comparisons involving more than two groups, the Kruskal–Wallis test was applied. The linear correlation analysis between miRNA expression and injury/clinical characteristics, was performed using Spearman’s test (correlation coefficient ρ [rho]). The statistical software GraphPad Prism 10 (Dotmatics, San Diego, CA, USA) was used for all statistical analyses. Results were considered statistically significant when *p* ≤ 0.05, and a *p*-value > 0.05 and <0.1 was rated as a statistical trend.

## 5. Conclusions

Our study demonstrates that acute spinal cord injury is associated with distinct and dynamic changes in circulating miRNA profiles. At admission, 46 miRNAs were differentially expressed (18 upregulated, 28 downregulated), followed by a global suppression pattern at 48 h post-injury, with 47 miRNAs downregulated. The pronounced suppression of miR-182-5p, miR-144-5p, and miR-30c-5p—compared with polytrauma patients—suggests SCI-specific mechanisms that may influence secondary injury processes through key molecular targets. Pathway analysis identified mitochondrial functions, including oxidative phosphorylation and electron transport chain components, as top enriched pathways, underscoring the critical role of oxidative stress in post-injury pathophysiology. While limited by sample size, these findings establish circulating miRNAs as promising biomarkers for early injury characterization and provide a molecular foundation for future studies aimed at improving diagnosis, prognosis, and therapeutic intervention in SCI.

## Figures and Tables

**Table 1:** Patient Demographics and Clinical Characteristics.

Characteristics	SCI (*n* = 8)	PT (*n* = 8)	*p*-Value
Male [n]	6	6	
Age [years] (IQR)	43.5 (32.3–66.5)	41 (35.5–50.5)	>0.999
ISS [points] (IQR)	25 (25–25)	25 (25–26)	0.446
IL-6 ER [pg/mL] (IQR)	23 (5.3–31.4)	60.8 (32.6–134)	0.094
IL-6 48h [pg/mL] (IQR)	30.9 (3.2–144)	101.3 (39.3–215)	0.187
Leukocytes ER [/nL] (IQR)	8.2 (5.7–10.2)	11.3 (8.2–22.3)	0.130
Leukocytes 48h [/nL] (IQR)	10.6 (6.8–13.3)	9.4 (5.7–10.3)	0.195
Hemoglobin ER [g/dL] (IQR)	12.4 (10.7–13.3)	12.1 (10–13.8)	0.879
Lactate ER [mg/dL] (IQR)	19.5 (13.8–39)	40.5 (27–52.3)	0.078
SBP at scene [mmHg] (IQR)	85 (72.5–117.5)	120 (112.5–137)	**0.038**
Catecholamines ER [%]	75	25	0.132
ICU LOS [days] (IQR)	8.5 (3.8–16.3)	5.5 (3.5–8.5)	0.267
Mechanical ventilation [days] (IQR)	2 (0.3–16)	2 (1–2)	0.694
in-hospital mortality [%]	0	0	

Abbreviations: ER—emergency room; ICU—intensive care unit; IL—interleukin; IQR—interquartile range (25–75%); ISS—injury severity score; LOS—length of stay; mmHg—millimeters of mercury; PT—polytrauma; SBP—systolic blood pressure; SCI—spinal cord injury. Significant *p*-values (*p* < 0.05) are shown in bold.

**Table 2:** Expression levels of candidate miRNAs in SCI patients and healthy controls at different time points.

miRNA	Healthy Control Mean * (SD)	SCI Mean (SD)	*p*-Value
**ER upregulated**			
hsa-miR-34a-5p	0.0035 (0.0038)	0.0084 (0.0091)	0.3807
hsa-miR-335-5p	0.0170 (0.0083)	0.0484 (0.0325)	**0.0379**
hsa-miR-193a-5p	0.0017 (0.0013)	0.0180 (0.0156)	**0.0006**
hsa-miR-450b-5p	0.0008 (0.0009)	0.0061 (0.0082)	**0.0145**
hsa-miR-582-3p	0.0010 (0.0010)	0.1430 (0.1658)	**0.0003**
**ER downregulated**			
hsa-miR-144-5p	0.0438 (0.0267)	0.0078 (0.0045)	**0.0003**
hsa-miR-30c-5p	0.1379 (0.0520)	0.0939 (0.0470)	0.1049
hsa-miR-182-5p	0.0186 (0.0114)	0.0018 (0.0007)	**0.0002**
hsa-miR-215-5p	0.0373 (0.0198)	0.0374 (0.0408)	0.3823
hsa-miR-190a-5p	0.0180 (0.0150)	0.0027 (0.0022)	**0.0011**
**48 h downregulated**			
hsa-miR-122-5p	0.0283 (0.0201)	0.0186 (0.0163)	0.4136
hsa-miR-215-5p	0.0373 (0.0198)	0.0110 (0.0114)	**0.0200**
hsa-miR-375-3p	0.0078 (0.0101)	0.0035 (0.0054)	0.1419
hsa-miR-150-5p	0.0879 (0.0700)	0.0441 (0.0397)	0.1079
hsa-miR-885-5p	0.0112 (0.0051)	0.0118 (0.0059)	>0.9999

* Values are presented as mean expression levels (normalized concentrations) with standard deviation (SD). Abbreviations: ER—emergency room; SCI—spinal cord injury; SD—standard deviation. *p*-values shown in bold indicate statistical significance. Significant *p*-values (*p* < 0.05) are shown in bold.

## Data Availability

The raw and processed miRNA expression data generated during this study have been deposited in the NCBI Gene Expression Omnibus (GEO) under accession number GSE309413. These data are publicly available at https://www.ncbi.nlm.nih.gov/geo/query/acc.cgi?acc=GSE309413 (accessed on 6 October 2025).

## Supplementary Materials

The following supporting information can be downloaded at: https://www.mdpi.com/article/10.3390/ijms262210954/s1.

## Abbreviations

The following abbreviations are used in this manuscript:

ASIA	American Spinal Injury Association
BCL	B-cell lymphoma
BMP	Bone morphogenetic protein
CCN	Cyclin
CNS	Central nervous system
CSF	Cerebrospinal fluid
ddPCR	Droplet digital polymerase chain reaction
ER	Emergency room
FDR	False discovery rate
FOX	Forkhead box
HC	Healthy control
ICU	Intensive care unit
IL	Interleukin
IQR	Interquartile range
I/R	Ischemia/reperfusion
miRNA	MicroRNA
NGS	Next-generation sequencing
Nrf2	Nuclear factor erythroid 2–related factor 2
ORA	Over representation analysis
OXPHOS	Oxidative phosphorylation system
PT	Polytrauma
ROS	Reactive oxygen species
SCI	Spinal cord injury
SD	Standard deviation
SMAD	Mothers against decapentaplegic homolog
STROBE	Strengthening the Reporting of Observational Studies in Epidemiology
TGF-β	Transforming growth factor beta
TNF-α	Tumor necrosis factor alpha
