# Supplementary material for: Circulating microRNA Profiles in Acute Spinal Cord Injury: Evidence for Distinct Plasma Signatures Compared with Polytrauma Patients
Source: Int J Mol Sci. 2025 Nov 12;26(22):10954. doi: 10.3390/ijms262210954 (PMC12652018; doi:10.3390/ijms262210954)
Supplement: Supplementary file 1 [file ijms-26-10954-s001.zip › ijms-3945878-supplementary.pdf]

Supplementary Data

Table S1. Primers for droplet digital PCR.

miRNA	Kit	Gene Globe ID	Catalog Nr.	Mature miRNA sequence
hsa-miR-375-3p	miRCURY LNA miRNA PCR Assay	YP00204362	339306	UUUGUUCGUUCGGCUCGCGUGA
hsa-miR-150-5p	miRCURY LNA miRNA PCR Assay	YP00204660	339306	UCUCCCAACCCUUGUACCAGUG
hsa-miR-144-5p	miRCURY LNA miRNA PCR Assay	YP00204670	339306	GGAUAUCAUCAUAUACUGUAAG
hsa-miR-30c-5p	miRCURY LNA miRNA PCR Assay	YP00204783	339306	UGUAAACAUCCUACACUCUCAGC
hsa-miR-182-5p	miRCURY LNA miRNA PCR Assay	YP00206070	339306	UUUGGCAAUGGUAGAACUCACACU
hsa-miR-215-5p	miRCURY LNA miRNA PCR Assay	YP00204598	339306	AUGACCUAUGAAUUGACAGAC
hsa-miR-190a-5p	miRCURY LNA miRNA PCR Assay	YP00204763	339306	UGAUAUGUUUGAUAUAUUAGGU
hsa-miR-34a-5p	miRCURY LNA miRNA PCR Assay	YP00204486	339306	UGGCAGUGUCUUAGCUGGUUGU
hsa-miR-335-5p	miRCURY LNA miRNA PCR Assay	YP02119293	339306	UCAAGAGCAAUAACGAAAAAUGU
hsa-miR-193a-5p	miRCURY LNA miRNA PCR Assay	YP00204665	339306	UGGGUCUUUGCGGGCGAGAUGA
hsa-miR-450b-5p	miRCURY LNA miRNA PCR Assay	YP00205607	339306	UUUUGCAAUAUGUUCCUGAAUA
hsa-miR-582-3p	miRCURY LNA miRNA PCR Assay	YP00204072	339306	UAACUGGUUGAACAACUGAACC
hsa-miR-122-5p	miRCURY LNA miRNA PCR Assay	YP00205664	339306	UGGAGUGUGACAAUGGUGUUUG
hsa-miR-885-5p	miRCURY LNA miRNA PCR Assay	YP00204473	339306	UCCAUUACACUACCCUGCCUCU

Primers for the indicated miRNAs, including catalog numbers and sequences, were obtained from Qiagen N.V. (Venlo, Netherlands).

Table S2. Upregulated plasma miRNAs at emergency room admission (0h) in SCI patients compared with healthy controls.

miRNA	log_2FC (SCI ER vs. HC)	p-value (SCI ER vs. HC)	score
hsa-miR-335-5p	1.772	1.59×10^{-6}	10.28
hsa-miR-193a-5p	2.361	8.65×10^{-5}	9.59
hsa-miR-450b-5p	2.419	2.60×10^{-4}	8.67
hsa-miR-214-3p	2.279	4.04×10^{-4}	7.73
hsa-miR-199b-3p	1.102	9.74×10^{-4}	3.32
hsa-miR-199a-3p	1.130	9.87×10^{-4}	3.40
hsa-miR-148a-3p	1.426	1.66×10^{-3}	3.97
hsa-miR-34a-5p	3.815	1.64×10^{-3}	10.63
hsa-miR-582-3p	3.085	2.65×10^{-3}	7.95
hsa-miR-424-5p	1.671	2.72×10^{-3}	4.29
hsa-miR-195-5p	1.812	5.99×10^{-3}	4.03
hsa-miR-143-3p	1.622	6.44×10^{-3}	3.56
hsa-miR-365b-3p	2.167	7.18×10^{-3}	4.65
hsa-miR-190b-5p	2.771	7.88×10^{-3}	5.83
hsa-miR-365a-3p	2.247	1.13×10^{-2}	4.37
hsa-miR-145-3p	1.595	1.19×10^{-2}	3.07
hsa-miR-452-5p	2.665	2.37×10^{-2}	4.33
hsa-miR-193b-3p	2.471	2.94×10^{-2}	3.79

Abbreviations: FC- fold change; HC- healthy controls; SCI- spinal cord injury; hsa- homo sapiens.

Table S3. Downregulated plasma miRNAs at emergency room admission (ER) in SCI patients compared with healthy controls.

miRNA	log_2FC (SCI ER vs. HC)	p-value (SCI ER vs. HC)	score
hsa-miR-30c-5p	-1.759	6.99×10^{-7}	-10.83
hsa-miR-144-5p	-2.918	4.37×10^{-6}	-15.64
hsa-let-7a-5p	-1.552	1.40×10^{-4}	-5.98
hsa-let-7d-5p	-1.405	1.88×10^{-4}	-5.23
hsa-miR-215-5p	-2.548	3.04×10^{-4}	-8.96
hsa-miR-23b-3p	-1.453	3.55×10^{-4}	-5.01
hsa-miR-182-5p	-3.050	7.29×10^{-4}	-9.57
hsa-miR-183-5p	-2.427	1.13×10^{-3}	-7.15
hsa-miR-190a-5p	-2.767	1.33×10^{-3}	-7.96
hsa-let-7f-5p	-1.356	1.59×10^{-3}	-3.80
hsa-miR-142-3p	-1.483	2.20×10^{-3}	-3.94
hsa-miR-144-3p	-2.176	2.63×10^{-3}	-5.61
hsa-miR-106a-5p	-1.863	4.86×10^{-3}	-4.31
hsa-miR-454-3p	-1.316	6.78×10^{-3}	-2.85
hsa-miR-30b-5p	-1.146	8.50×10^{-3}	-2.37
hsa-miR-103b	-1.093	1.00×10^{-2}	-2.19
hsa-miR-103a-3p	-1.036	1.10×10^{-2}	-2.03
hsa-miR-375-3p	-1.685	1.11×10^{-2}	-3.29
hsa-miR-486-5p	-1.388	1.12×10^{-2}	-2.72
hsa-miR-451a	-1.624	1.18×10^{-2}	-3.13
hsa-miR-486-3p	-1.477	1.48×10^{-2}	-2.70
hsa-let-7g-5p	-1.036	1.95×10^{-2}	-1.77
hsa-miR-9-5p	-3.712	2.22×10^{-2}	-6.14
hsa-let-7b-5p	-1.024	3.02×10^{-2}	-1.56
hsa-miR-18a-5p	-1.066	3.75×10^{-2}	-1.52
hsa-miR-106b-5p	-1.111	4.20×10^{-2}	-1.53
hsa-miR-16-5p	-1.095	4.39×10^{-2}	-1.49
hsa-miR-363-3p	-1.358	4.85×10^{-2}	-1.79

Abbreviations: FC- fold change; HC- healthy controls; SCI- spinal cord injury; hsa- homo sapiens.

Table S4. Downregulated plasma miRNAs at 48 hours post-injury in SCI patients compared with healthy controls.

miRNA	log_2FC (SCI ER vs. HC)	p-value (SCI ER vs. HC)	score
hsa-miR-122-5p	-3.606	2.71×10^{-7}	-23.68
hsa-miR-150-5p	-2.047	1.87×10^{-7}	-13.77
hsa-miR-215-5p	-3.254	9.92×10^{-6}	-16.28
hsa-miR-30c-5p	-1.540	1.63×10^{-5}	-7.37
hsa-miR-122b-3p	-4.767	1.72×10^{-5}	-22.71
hsa-miR-375-3p	-3.044	2.06×10^{-5}	-14.26
hsa-miR-23b-3p	-1.559	1.47×10^{-4}	-5.97
hsa-miR-192-5p	-2.428	1.50×10^{-4}	-9.28
hsa-miR-342-3p	-1.374	5.20×10^{-4}	-4.51
hsa-miR-885-5p	-3.681	6.90×10^{-4}	-11.64
hsa-miR-144-5p	-2.030	1.37×10^{-3}	-5.81
hsa-miR-194-5p	-2.239	2.06×10^{-3}	-6.02
hsa-miR-214-3p	-2.671	2.46×10^{-3}	-6.97
hsa-let-7d-5p	-1.137	2.53×10^{-3}	-2.95
hsa-let-7a-5p	-1.177	3.90×10^{-3}	-2.84
hsa-miR-486-5p	-1.552	4.51×10^{-3}	-3.64
hsa-miR-92a-3p	-1.258	4.69×10^{-3}	-2.93
hsa-miR-103a-3p	-1.143	5.39×10^{-3}	-2.59
hsa-miR-103b	-1.171	6.16×10^{-3}	-2.59
hsa-miR-100-5p	-1.665	6.47×10^{-3}	-3.64
hsa-miR-183-5p	-2.021	7.03×10^{-3}	-4.35
hsa-let-7b-5p	-1.264	7.49×10^{-3}	-2.69
hsa-miR-483-5p	-1.744	1.07×10^{-2}	-3.44
hsa-miR-15b-5p	-1.014	1.19×10^{-2}	-1.96
hsa-miR-99a-5p	-1.776	1.11×10^{-2}	-3.47
hsa-miR-10b-5p	-1.381	1.17×10^{-2}	-2.67
hsa-miR-30b-5p	-1.093	1.22×10^{-2}	-2.08
hsa-miR-182-5p	-2.263	1.27×10^{-2}	-4.33
hsa-miR-29b-3p	-1.517	1.45×10^{-2}	-2.79
hsa-miR-486-3p	-1.485	1.46×10^{-2}	-2.73
hsa-miR-29a-3p	-1.184	1.49×10^{-2}	-2.16
hsa-miR-451a	-1.546	1.65×10^{-2}	-2.76
hsa-miR-195-5p	-1.871	1.69×10^{-2}	-3.32
hsa-let-7g-5p	-1.051	1.79×10^{-2}	-1.84
hsa-miR-15a-5p	-1.085	2.22×10^{-2}	-1.79
hsa-miR-145-5p	-1.059	2.31×10^{-2}	-1.73
hsa-miR-144-3p	-1.469	4.27×10^{-2}	-2.01
hsa-miR-125b-5p	-1.139	4.82×10^{-2}	-1.50
hsa-miR-16-5p	-1.264	2.01×10^{-2}	-2.15
hsa-miR-10a-5p	-1.022	4.17×10^{-2}	-1.41
hsa-miR-106b-3p	-1.188	3.70×10^{-2}	-1.70
hsa-miR-190a-5p	-1.997	2.04×10^{-2}	-3.38
hsa-miR-6721-5p	-3.382	4.15×10^{-2}	-4.67
hsa-miR-454-3p	-1.132	2.17×10^{-2}	-1.88
hsa-miR-342-5p	-2.284	3.09×10^{-2}	-3.45
hsa-miR-25-3p	-1.187	2.20×10^{-2}	-1.97
hsa-miR-19a-3p	-1.058	1.88×10^{-2}	-1.83

Abbreviations: FC- fold change; HC- healthy controls; SCI- spinal cord injury; hsa- homo sapiens.

Table S5. Plasma concentrations of candidate miRNAs in SCI and polytrauma patients and healthy controls (HC) at different time points.

miRNA	HC vs. SCI ER means* *p-value*	HC vs. PT ER means *p-value*	SCI vs. PT ER means *p-value*	HC vs. SCI 48h means *p-value*	HC vs. PT 48h means *p-value*	SCI vs. PT 48h means *p-value*
miR-335-5p	0.0170	0.0170	0.0484	0.0170	0.0170	0.0197
	0.0484	0.1488	0.1488	0.0197	0.0214	0.0214
	0.0379	*0.0650*	*0.5737*	*0.8518*	*0.9591*	*0.8518*
miR-193a-5p	0.0017	0.0017	0.0180	0.0017	0.0017	0.0022
	0.0180	0.0965	0.0965	0.0022	0.0054	0.0054
	0.0006	***0.0003***	*0.1605*	*0.9497*	*0.0830*	*0.1812*
miR-450b-5p	0.0008	0.0008	0.0061	0.0008	0.0008	0.0062
	0.0061	0.0121	0.0121	0.0062	0.0014	0.0014
	0.0145	***0.0204***	*0.7984*	*0.0569*	*0.1866*	*0.1419*
miR-582-3p	0.0010	0.0010	0.1430	0.0010	0.0010	0.0177
	0.1430	0.3757	0.3757	0.0177	0.0271	0.0271
	0.0003	***0.0019***	*0.7984*	***0.0007***	***0.0003***	*0.5728*
miR-144-5p	0.0438	0.0438	0.0078	0.0438	0.0438	0.0053
	0.0078	0.0159	0.0159	0.0053	0.0074	0.0074
	0.0003	***0.0148***	0.3823	***0.0007***	***0.0019***	0.6620
miR-30c-5p	0.1379	0.1379	0.0939	0.1379	0.1379	0.0896
	0.0939	0.2873	0.2873	0.0896	0.0952	0.0952
	0.1049	*0.5054*	*0.1605*	*0.0813*	*0.1049*	*0.4908*
miR-182-5p	0.0186	0.0186	0.0018	0.0186	0.0186	0.0023
	0.0018	0.0062	0.0062	0.0023	0.0027	0.0027
	0.0002	***0.0281***	*0.0650*	***0.0007***	***0.0019***	*0.3450*
miR-215-5p	0.0373	0.0373	0.0374	0.0373	0.0373	0.0110
	0.0374	0.7253	0.7253	0.0110	0.0276	0.0276
	0.3823	***0.0104***	***0.0030***	***0.0200***	*0.1304*	*0.6620*
miR-190a-5p	0.0180	0.0180	0.0027	0.0180	0.0180	0.0023
	0.0027	0.0056	0.0056	0.0023	0.0040	0.0040
	0.0011	***0.0281***	*0.2345*	***0.0027***	***0.0148***	*0.9497*
miR-375-3p	0.0078	0.0078	0.0058	0.0078	0.0078	0.0035
	0.0058	0.0472	0.0472	0.0035	0.0022	0.0022

	0.7984	***0.0499***	***0.0148***	*0.1419*	***0.0499***	*0.9497*
miR-150-5p	0.0879	0.0879	0.1004	0.0879	0.0879	0.0441
	0.1004	0.1733	0.1733	0.0441	0.0562	0.0562
	0.3282	*0.2345*	*0.5737*	*0.1079*	*0.2345*	*0.5728*

*Values are presented as mean expression levels (normalized concentrations), measured by droplet digital PCR. Abbreviations: ER- emergency room; HC- healthy controls; SCI- spinal cord injury. p-values shown in bold indicate statistical significance.

Table S6. Target genes of candidate miRNAs based on miRTarBase.

miRNA	targets
hsa-miR-182-5p	**FOXO3**, **BCL2**, **CCND2**, **SMAD4**, ADCY6, MITF, RARG, CREB1, MTSS1, FGF9, NTM, CYLD, PFN1, RECK, FOXF2, PTEN, ZFAND4, SATB2, FLOT1, CHL1, LRRC4, PDCD4, NDRG1, THBS1, ULBP2, TCEAL7, FBXW7, PDK4, TRIM8, BDNF, PCDH8, NRN1, UQCRFS1
hsa-miR-144-5p	**SMAD4**, **CCNE1**, **CCNE2**, ROCK1, ROCK2, MET
hsa-miR-30c-5p	**FOXO3**, **CCND2**, **MCL1**, **BCL9**, **SMAD1**, UBE2I, SERPINE1, SNAI1, HSPA4, VIM, MTA1, SNAI2, CASP3, MTTP, NOTCH1, RASAL2, JAK1, EIF2S1, BECN1, CAMK2D, IER2, CTGF

Predicted target genes of candidate miRNAs based on miRTarBase. Genes shown in bold indicate either direct overlaps between different miRNAs or belong to the same gene family (e.g., BCL family, CCN family) identified during the target analysis.

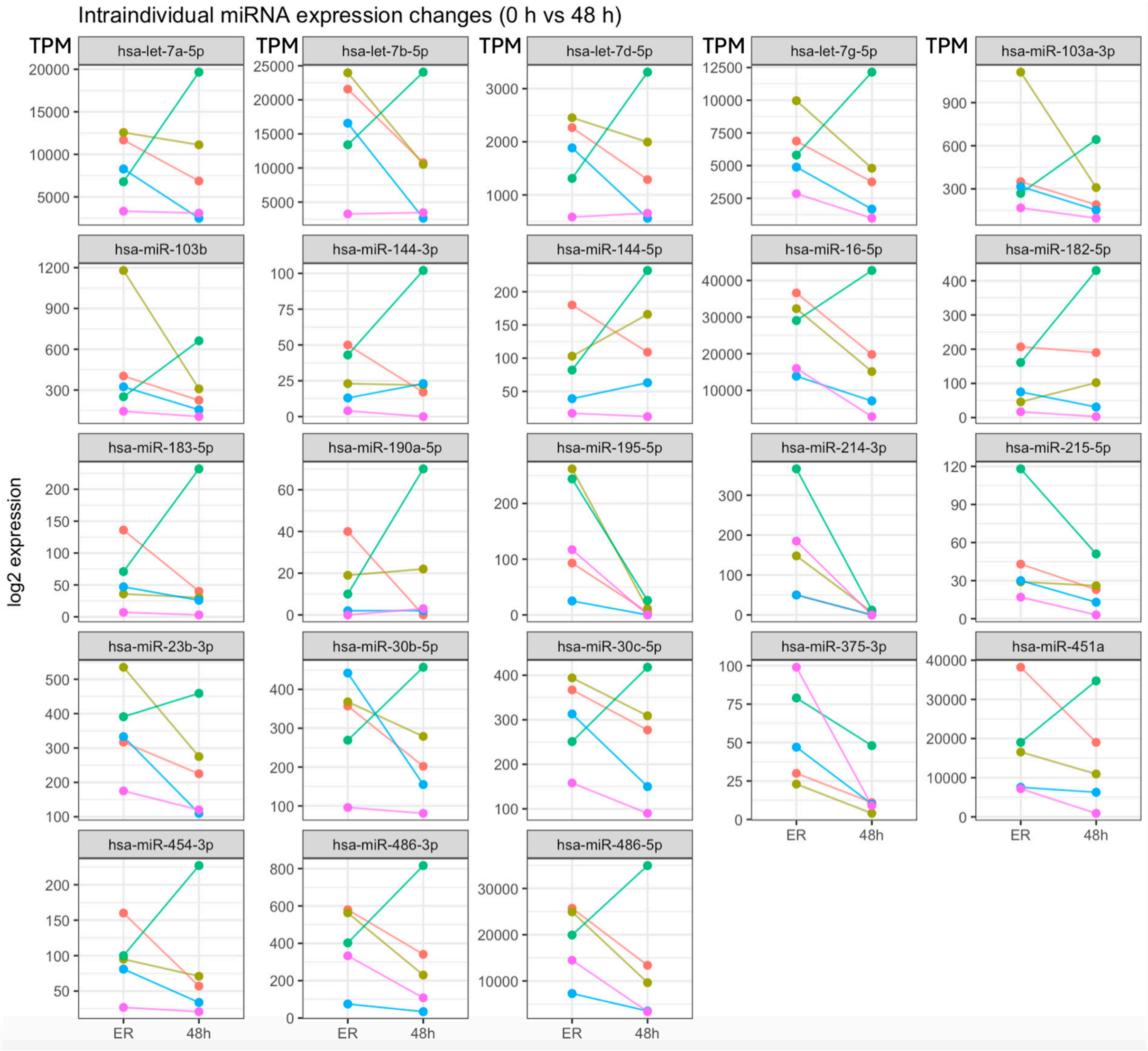

Figure S1. Intraindividual miRNA expression changes in patients with acute spinal cord injury (0 h vs. 48 h). Paired line plots illustrating intraindividual expression changes of 23 miRNAs detected at both time points (emergency room admission [ER, 0 h] and 48 h post-injury) in five SCI patients. Each line represents one individual patient. Expression values are shown as log_2-transformed transcripts per million (TPM) derived from small RNA sequencing. Most miRNAs demonstrated a consistent downward trend between ER and 48 h, suggesting that post-injury downregulation may represent a recurring, though variable, response pattern following acute SCI.
